# Supplementary material for: Sharp zero-phonon lines of single organic molecules on a hexagonal boron-nitride surface
Source: Nat Commun. 2023 Dec 2;14:7960. doi: 10.1038/s41467-023-42865-4 (PMC10693553; doi:10.1038/s41467-023-42865-4)
Supplement: Supplementary file 1 — Supplementary Information [file 41467_2023_42865_MOESM1_ESM.pdf]

Supplementary Information for

Sharp zero-phonon lines of single organic molecules on a hexagonal boron-nitride surface

Robert Smit[a], Arash Tebyani[a], Jil Hameury[a], Sense Jan van der Molen[a] and Michel Orrit[a,*]

[a] Huygens-Kamerlingh Onnes Laboratory, LION, Postbus 9504, 2300 RA Leiden, The Netherlands
[*] E-mail: Orrit@Physics.LeidenUniv.nl

The supplementary information contains additional data that was not included in the main text. In the main text, we frequently refer to the various sections and figures that are part of this document.

Supplementary Methods

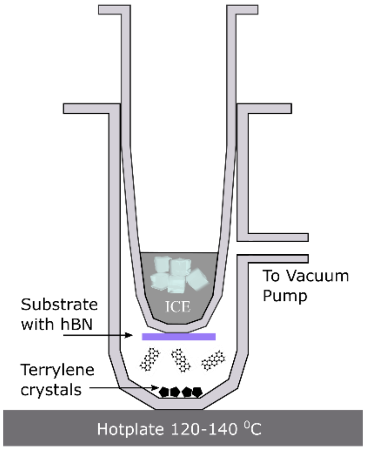

Supplementary Figure 1. a) Schematic representation of the sublimation apparatus, which consists of two round-bottom flasks, where the top one is inserted into the bottom one, sealed by vacuum grease (Apiezon) and pumped by a vacuum pump from the side. The bottom of the flask is heated on a hot plate to approximately 120-140 °C. The crystals are placed inside, in contact with the heated bottom. The sample is fixed with carbon tape on the cold finger and cooled with water ice.

Supplementary Notes 1. Room temperature measurements

AFM profiles

AFM characterization of the exfoliated hBN flakes shows that typical thicknesses are spread out over a broad range from 40 nm up to more than 300 nm, though in general they are below 100 nm in thickness. Planar sizes are typically at least 10 µm x 10 µm, but never more than a few hundred µm in diameter. In some cases, wrinkles can be observed in the sheet (Figure 2d). Figures 2e and 2f show the same flake before and after the annealing process (500 ˚C for 12 h). Most noticeable is that the contamination/tape residue outside of the flake area are efficiently removed by the annealing process.

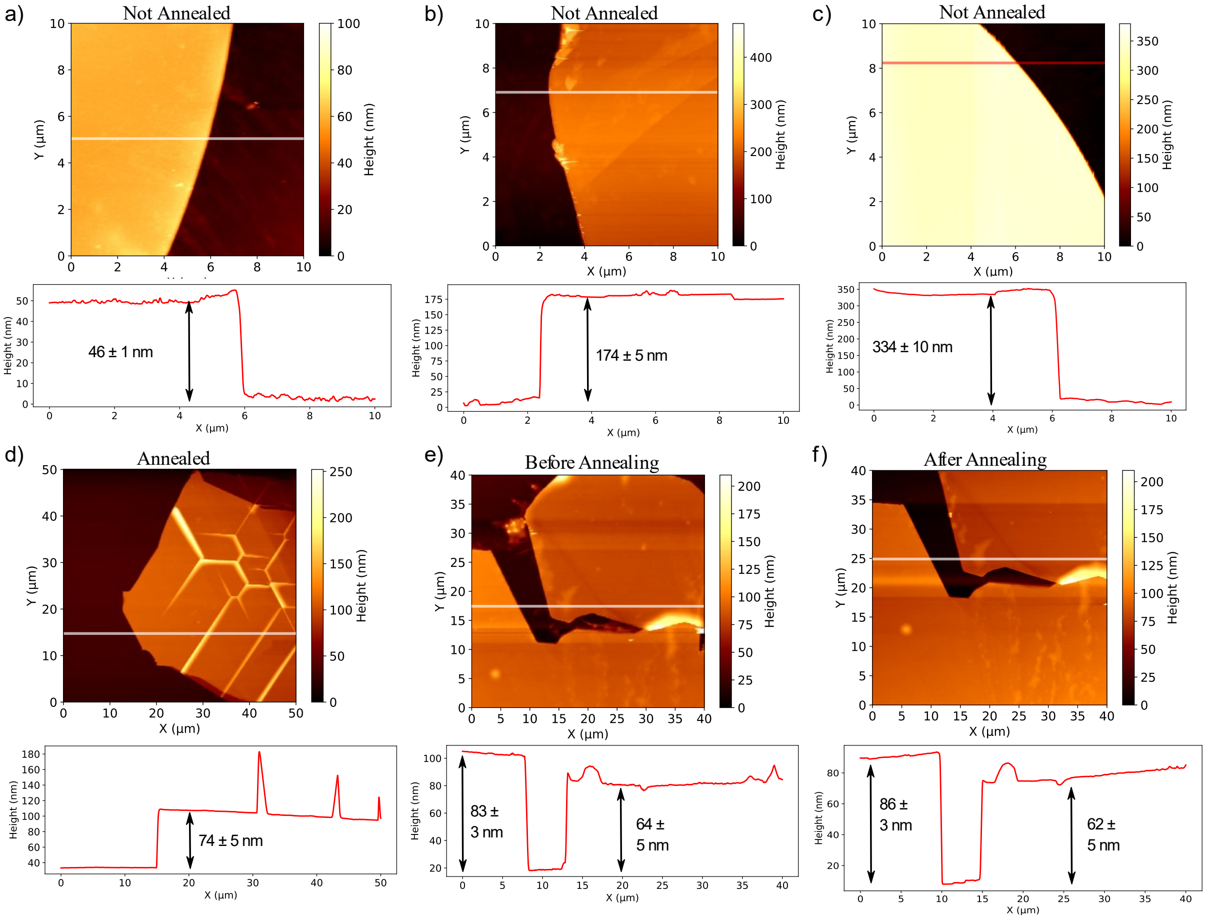

Supplementary Figure 2. a,b,c) AFM scans of a small part (10x10 µm^2) of non-annealed exfoliated hBN flakes on a Si/SiO$_2$ substrate with under each 2D map the corresponding height profile along the white line (red in c). The thickness of hBN flakes was found to vary significantly from flake to flake, ranging from 46 up to 334 nm, but typically below 100 nm. The flake in (d) was annealed at 500 ˚C for 12 hours. The AFM images in (e) and (f) show the same flake, respectively before and after annealing at 500 ˚C for 12 hours. Tape residues or other contaminants on the substrate, outside of the flakes, are efficiently removed by annealing. On the flakes themselves there is no significant difference. Possibly some contamination on the substrate in the bottom right corner, protected by the hBN flake, does not get removed by annealing. The AFM image in (d) shows clear wrinkles in the hBN sheet, appearing as tall peaks in the AFM profile.

The fluorescence image in Figure 3a shows the hBN flake measured by AFM before and after annealing (Figures 2e, 2f). We deposited terrylene on this flake after annealing by sublimation at an increased rate, by heating to 135-140 ˚C for 5 minutes. Surprisingly, the number of visible emitters is much lower than for the non-annealed flake in Figure 1a of the main text. We systematically find less molecules on the annealed flakes, than on non-annealed flakes. Although there are fewer molecules, there are some locations with higher densities of emitters, which seem to have more surface roughness (Figure 3b). Fewer emitters are typically found on the more pristine parts of the flake, which can be near-to atomically flat (Figure 4).

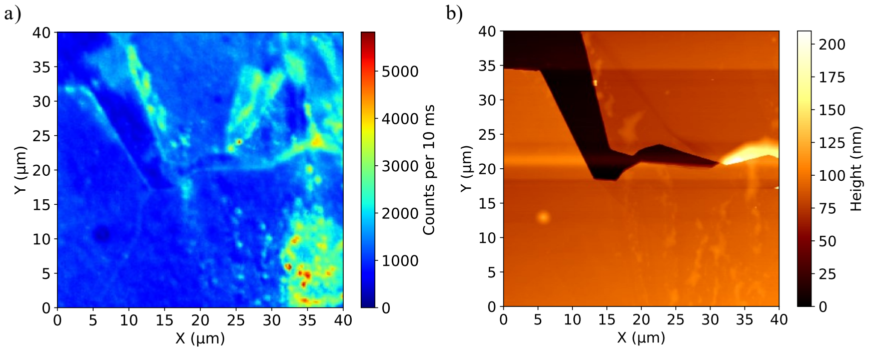

Supplementary Figure 3. a) Fluorescence image of the same area that we measured with AFM in Figure 2e and 2f, before and after annealing. For comparison, the latter AFM image, after annealing, is recalled in b. The flake was annealed at 500 ˚C for 12 h. Even though we increased the sublimation temperature to 135-140 ˚C, the number of visible emitters is clearly much less than for example in the non-annealed flake in Figure 1a of the main text. Terrylene molecules are mostly found in the bottom right corner of the image. In this region, the AFM profile shows more surface roughness, which might be caused by contamination under the hBN layer. The flatter regions of the hBN flake show less fluorescence. The AFM measurement was done before deposition of the molecules and subsequent low-temperature experiments. The region in (a) for 25 < X < 30 µm and 23 < Y < 35 µm appears to have changed with respect to the same region in b), perhaps due to a folding of a part of the flake by the AFM tip.

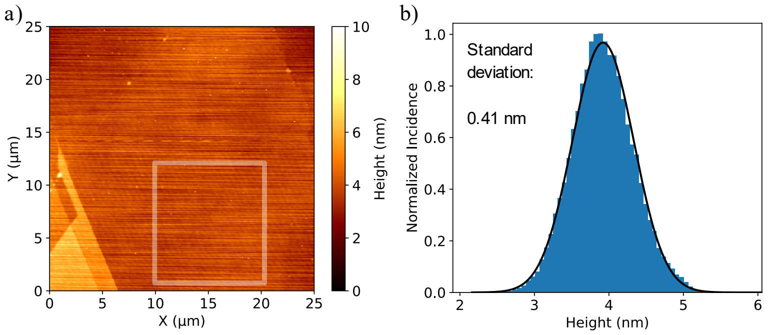

Supplementary Figure 4. a) AFM image of a flat region on an annealed hBN flake (500 ˚C for 12 h). The white rectangle encloses the region that builds up the histogram of surface heights in b). The Gaussian distribution of surface heights shows a standard deviation of about 0.41 nm. Tilts and drifts in the scan were subtracted by a line-by-line linear fit.

Room temperature spectra of terrylene

The broadness of the spectra of single emitters on hBN at room temperature makes it difficult to identify their origin, due to a lack of spectral features. Moreover, typical defect emitters in hBN contain an emission band shifted about 1,365 cm^{-1} from the main emission peak. This is due to the emitter's coupling to the B-N stretch vibrational mode, which is Raman active as shown in Figure 5. The B-N stretch is very close to the C-C stretch modes of polyaromatic hydrocarbons, which typically lie around 1,200-1,300 cm^{-1}. Hence, the distinction between various emitters present in hBN itself, impurities of the toluene and finally the terrylene itself was inherently difficult.

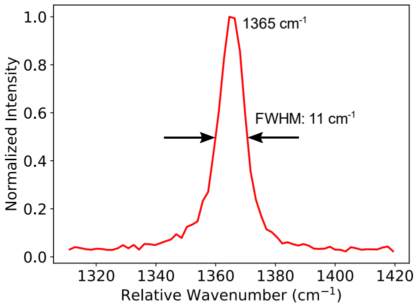

Supplementary Figure 5. Spectrum of the inelastically scattered light from a multilayer hBN flake with a 532 nm excitation wavelength. The single Raman peak observed at 1365 cm^{-1} with a FWHM of 11 cm^{-1}, is significantly broader than the

spectrograph resolution, around 1-2 cm^{-1}. The spectrum is obtained at room temperature with a 1200 lines/mm grating and a 0.1 mm slit size. The position and width of the Raman peak conforms with the data provided by the supplier hq-graphene.

For terrylene in general, we find molecules whose spectra are broadened in such a way that no single vibrations can be resolved, as different modes merge into broad bands. A common spectrum of a terrylene molecule on hBN looks like the spectrum in Figure 6a. The strongest peak around 580 nm is a superposition of the phonon-broadened purely electronic transition and of the main low-frequency vibration around 245-250 cm^{-1}. The second band around 630 nm is dominated by a bunch of C-C stretch modes with first overtones around 680 nm. On some of the annealed samples, we find molecules that are less broadened, such as in Figure 6b. In this spectrum, a more detailed structure of the vibrations is revealed with a higher-resolution grating (600 lines/mm). Consistent with the low temperature results, we find most of the molecules to peak around 580 nm.

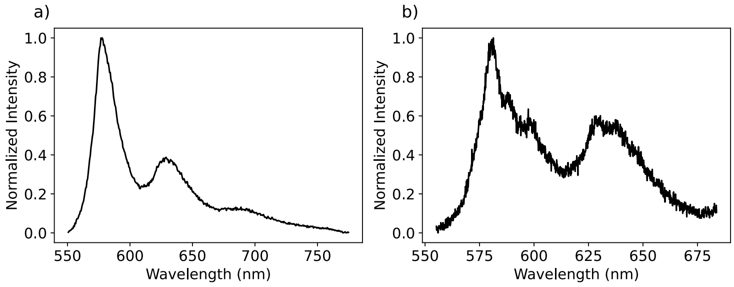

Supplementary Figure 6. Emission spectra of terrylene on hBN at room temperature, recorded with a 150 lines/mm grating for the spectrum in a) and a 600 lines/mm grating in b). The spectra could stem from ensembles of molecules ($g^{(2)}(\tau)$ was not measured), which may lead to additional inhomogeneous broadening.

Adsorption of terrylene on hBN

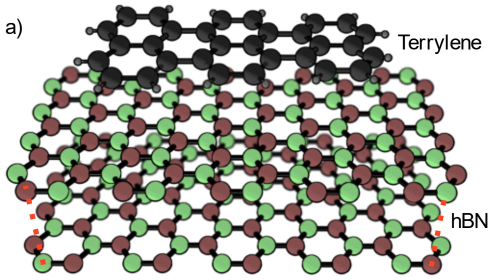

Supplementary Figure 7. Schematic perspective view of terrylene on hBN, with brown colors displayed as boron and green as nitrogen. As the real adsorption sites for terrylene on hBN are unknown, we assume that, similar to pentacene at monolayer coverage[1,2], terrylene lies flat on the hBN substrate, bonded by weak Van der Waals forces. The hBN monolayers stack with boron facing nitrogen atoms at adjacent planes (dashed red lines).

Terrylene in isolated form has a practically flat structure (see Figure 7). In some matrices terrylene was found to exhibit a slight twist of a few degrees of the outer naphthalene units around the central naphthalene unit according to quantum chemistry calculations[3,4]. The interatomic distance of nitrogen and boron in the hexagonal lattice of hBN is 1.45 Å[5] and matches well within about 7% of the C-C bond lengths of the terrylene molecule[6]. In addition, the interlayer distance between monolayer sheets, about 3.33 Å, is practically identical in graphite and hBN crystals[7]. Therefore we expect the terrylene molecule, itself in approximation a small patch of graphene terminated by hydrogen atoms, to lie flat on the surface of hBN, bound by weak Van der Waals interactions, which are also responsible for the stacking of hBN or graphene monolayers and even hBN/graphene heterostructures[8]. Pentacene, a molecule comparable to terrylene, was indeed found to lie flat on the surface of graphene[9] and of hBN as a monolayer[1,2].

Unlike in graphene, the alternating arrangement of nitrogen and boron in hBN opens up a band gap, which was measured to be 6.08 eV for the bulk crystal using two-photon excitation spectroscopy[10]. The large band gap prevents the exchange of electrons or energy with terrylene, which has a HOMO-LUMO gap between the singlet ground and excited state in the range of 2.1 ± 0.1 eV[11]. In fact, the band gap of hBN is sufficiently large to probably prevent any exchange of electrons or energy with basically all known fluorescent dyes used in single-molecule spectroscopy.

Supplementary Notes 2. Comparison of terrylene spectra on hBN and other matrices

Below we list the vibrational frequencies measured in Figure 2c and 2d in the main text with an assignment of the specific vibrations as reported in the literature and a comparison to terrylene in a few three-dimensional matrices. The positions of the vibrational frequencies and the number of lines may however vary slightly from molecule to molecule.

Supplementary Table 1. Frequencies (in cm^{-1}) and assignments of the vibrations of the terrylene molecules in the main site at 582 nm and the red site at 602 nm, as shown in Figure 2c and 2d in the main text. The vibrational frequencies are compared with terrylene measured in three types of matrices, respectively a semi-crystalline polymer, a Shpol'skii matrix and an aromatic matrix.

Assignment[6,12]	hBN (main site): 582 nm	hBN (red site): 602 nm	Polyethylene[12]	*n*-Hexadecane[3]	Anthracene[13]
0-0 Zero-phonon line	0	0	0	0	0
Long-axis stretching	246	251	242	241	247
	437	439? (very weak)	442	439	
2 × 246	494	501	487		496
In-plane ring deformation	534	536	534	532	536
	580	582	584	581	584
	684				
3 × 246	739		736		
534 + 246	780		780		
	828	847?	830		843
Aromatic C=C stretch	1259	1265	1269	1269	
	1276		1280	1279	1278
	1303	1290	1297	1309	1292
	1349		1355	1357	
	1359				
	1366	1362			1366
1259 + 246	1508		1529	1506	
1276 + 246	1527			1522	
	1555	1562	1556	1553	1566
	1601		1580		
1555 + 246	1801		1802		1815

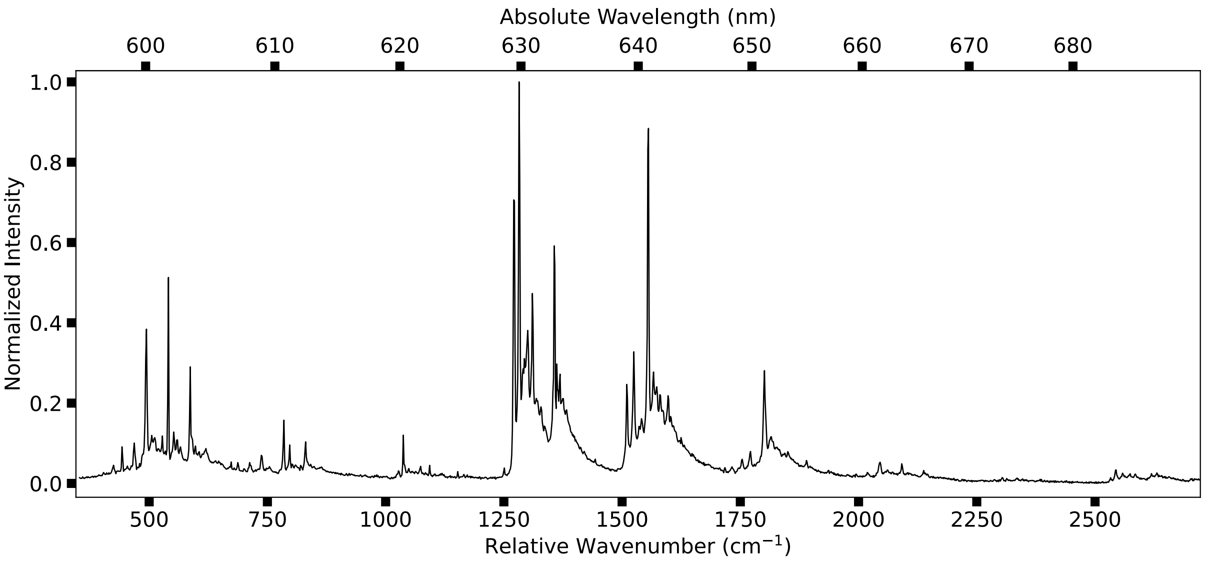

Supplementary Figure 8. Emission spectrum from the resonantly-excited molecule in Figure 5 in the main text. The excitation wavelength was 582.38 nm and we used a 590 Long pass filter to block the laser light. Consequently, the 0-0 ZPL and main vibrational peak is not visible. The spectrum was recorded for 150 s using a 1,200 lines/mm grating and a 50 µm slit size. T = 2 K.

Supplementary Notes 3. Thermal broadening of the 0-0 ZPL linewidth

We measured the 0-0 ZPL linewidths in Figure 2b in the main text by recording the spectra of a single molecule located on an annealed hBN flake (750 ˚C for 12 h). A subset of 9 out of the 88 different temperatures at which we recorded spectra, is shown in Figure 9. A weak contribution from another emitter around 579-580 nm is observed in the spectrum at 2 K. The relative intensities of the two emitters varied due to drifts in the laser spot as the temperature changed. To account for these drifts, we realigned the microscope to the main molecule at intervals of about 5 K.

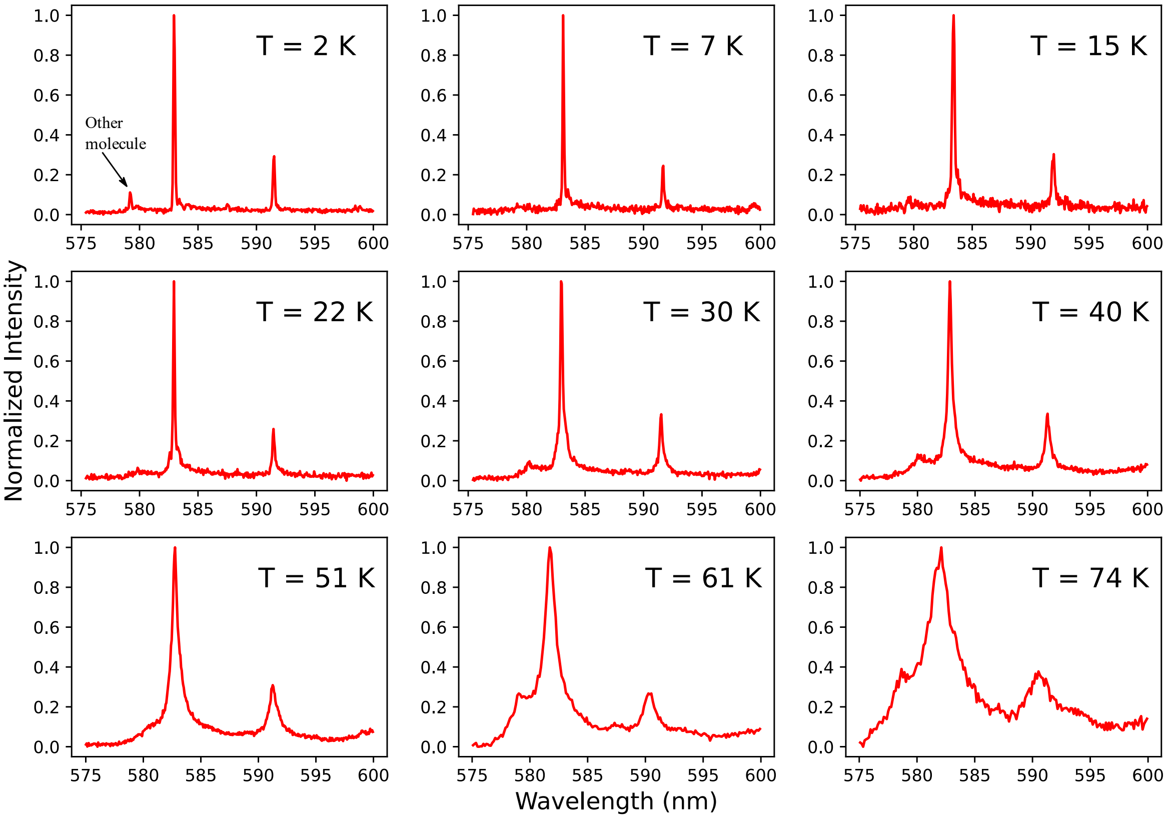

Supplementary Figure 9. Nine emission spectra recorded at intermediate temperatures in a range from 2 K up to 74 K. From the main peak around 583 nm, we extract the linewidth from the full-width-at-half-maximum, in order to build up Figure 2b in the main text. A weak line of another emitter is present around 579 nm (indicated by arrow in top left figure). The first five spectra were integrated for 10 seconds, while the remaining spectra were integrated for 30 seconds. As the spectral lines broaden with temperature, the fluorescence intensity will be distributed over more pixels. Hence, with a longer integration we still obtained a relatively good signal per pixel.

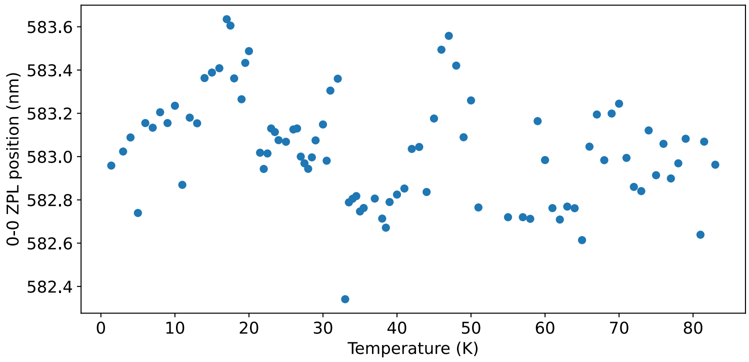

Supplementary Figure 10. 0-0 ZPL position with temperature, determined from the 88 spectra that built up Figure 3b in the main text. The spectral position of the 0-0 ZPL may vary due to spectral jumps, which are indeed present as observed in Figure 11. Overall, the 0-0 ZPL position shows no temperature-induced shift or trend in the measured temperature range.

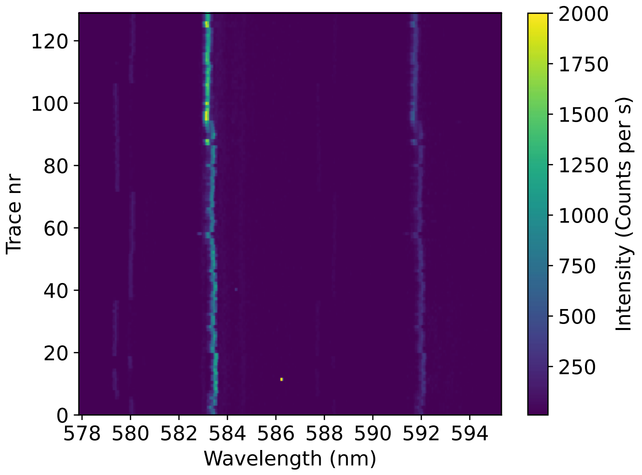

Supplementary Figure 11. Time series of spectra of the molecule that we measured for the broadening of the 0-0 ZPL with temperature in Figure 2b in the main text. There are spectral jumps present that we attribute to the fluctuations in the main 0-0 ZPL position in Figure 10. Possibly, these fluctuations may add uncertainty to the points in Figure 2b in the main text, as the relatively long integration time of 10-30 s per spectrum may broaden the 0-0 ZPL by spectral jumps occurring within that time frame. The emitter appearing around 579-580 nm, as observed for the spectra in Figure 9, is also present in this series and shows clear coupling to a two-level system. T = 2 K.

Supplementary Notes 4. Spectral wandering

Spectral instabilities of the single molecules are always present on our samples, in the form of clear jumps between two levels up to very complex jittering of the spectral position of the molecule. In this section we will show results of experiments we performed to characterize and find the source of the spectral diffusion.

Photo-induced spectral jumps

As we describe in the main text, the spectral diffusion is photo-induced at constant temperature. We confirmed this by running a series of spectra, while closing the shutter for two short periods (Figure 12). After the shutter was opened again, the molecules continued the trace at the position where they left.

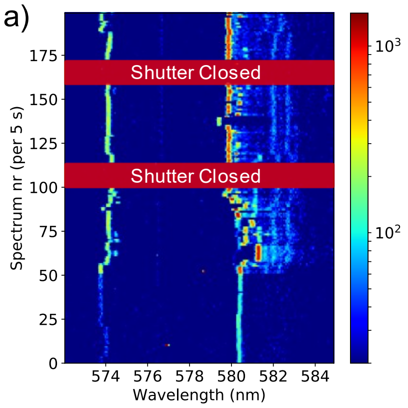

Supplementary Figure 12. Spectral time trace of the 0-0 zero-phonon lines of a couple of molecules that are in the focal spot. For the first 50 traces the excitation intensity is 6 kW/cm^2 and the remainder is recorded with a 60 kW/cm^2 excitation intensity: the excitation intensity was increased to have a faster rate of spectral jumps and increase the number of visible molecules. The red regions indicate periods during which the shutter for the excitation path was closed. Once the shutter is re-opened, the molecules indeed continue from the same spectral position they had before the shutter was closed. T = 2 K.

Spatial diffusion

As hBN is known to be an excellent lubricant, we considered that the molecule might 'skate' over the surface at room temperature, or perhaps even at low temperature: spatial diffusion. Such a spatial diffusion might involve rotations of the molecule, which we can explicitly track by following the polarization of the molecule's fluorescence. The polarization of fluorescence is determined by the transition dipole moment vector, which for terrylene is oriented along the long axis of the molecule. To track changes in the polarization of the fluorescence, we installed a polarizing beam splitter in our setup, to separate the s-polarization and p-polarization components. We followed both polarization channels at the outputs of the beam splitter by recording the fluorescence signals with an APD. Simultaneously, we recorded spectra of the molecule, in order to detect spectral jumps. When a molecule rotates, the signals are expected to redistribute over the two detectors, i.e. show anticorrelated fluctuations. In Figure 13 we trace the fluorescence signal of the two polarization channels, with corresponding spectra of the molecule on the right side. For the second molecule in particular (c, d), there is a clear correlation between the two channels. Hence, the spectral jumps are not caused by rotation of the molecule.

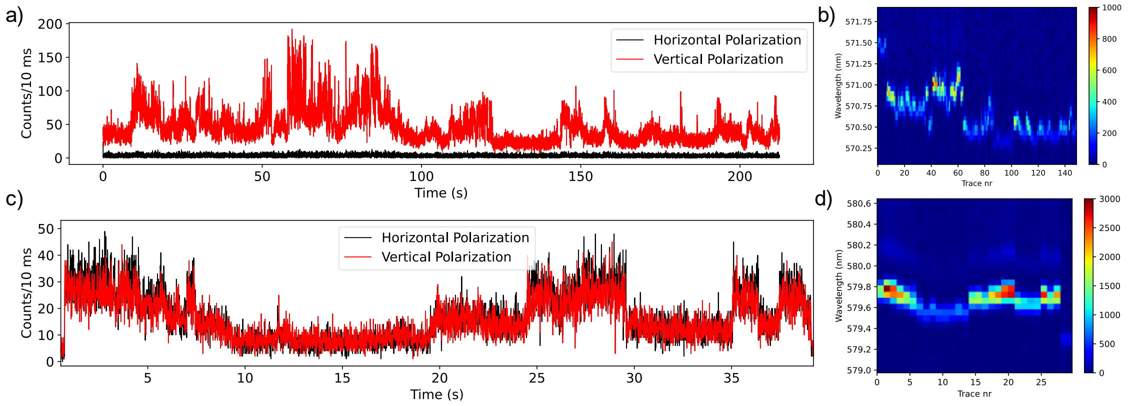

Supplementary Figure 13. a,b) Fluorescence time trace and spectra of molecule A, which is nearly perfectly oriented with the vertical polarization component. Spectral jumps during the time trace have no influence on the horizontal polarization component. c,d) Shows the time trace and spectra of another molecule (molecule B) with comparable intensities in both polarization channels. Also here the spectral jumps have no influence on the ratio of the two polarization components. Around 28 s, molecule B jumps to a spectral position where the excitation was much less efficient, the signal dropped considerably, and therefore the data was cut here. The spectra in b) and d) were recorded with a 1 s integration time. The time scales of the fluorescence time trace and of the spectra are different, due to some delay between the recorded spectra. T = 2 K.

Even though there is no rotational diffusion, there is still a possibility of a translational motion. Below the diffraction-limit of our setup, translational motion could be detected using super-resolution imaging. As the molecule itself is a point source, compared to a diffraction-limited focal point, the position of the molecule can be deconvoluted by fitting the point-spread function (PSF) of the molecule by a 2D Gaussian of the form:

$$G(x, y) = B + A * e^{-((x-x_0)^2 + (y-y_0)^2)/\Sigma} \quad (1)\ .$$

Here, B is defined as the background, A the amplitude of the PSF, x_0 and y_0 the coordinates of the molecule in the focal plane and Σ a parameter characterising the spread of the PSF, assumed to be equal for the x and y direction. To measure the PSF of the molecule, we scanned the laser beam over the imaging plane. With more than two molecules present in the scan, we can trace the average distance between them by fitting both PSFs of the molecules to equation 1. An example of the experimental data and fit is shown in Figure 14. Both the integration time and the pixel width will influence the resolution of the molecule localization. To increase resolution, we programmed a 50 nm pixel size, with an integration time of 20 ms per pixel. Despite the high resolution of the scan and relatively high number of counts, the localization will be influenced by fluctuations of the fluorescence signal caused by spectral jumps.

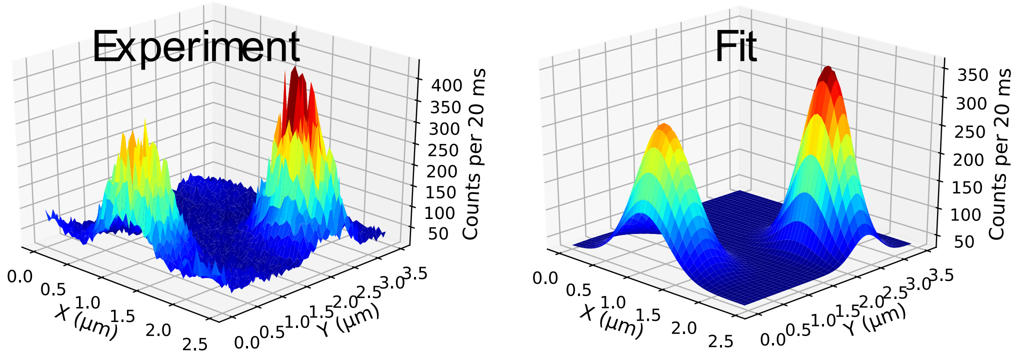

Supplementary Figure 14. Point-spread functions of two molecules that are approximately 2 µm apart from each other and do not (or barely do) overlap with the PSFs from other molecules. The left image shows the experimental data and the right image is the fit to the experimental data. A single scan consisted of 100x100 pixels over a range of 5x5 µm^2 and took about 200 s to acquire with the scanning mirror. In total, 24 of these images were acquired over the course of 90 minutes with short pauses in between. T = 2 K.

The relative distance of the two molecules in the set of images taken over a period of 90 minutes, as deviations from the mean distance, are shown in Figure 15. Despite the relatively high number of counts, the resolution may be in particular limited due to spectral jumps. With a fitted FWHM (full width at half maximum) of approximately 700-800 nm (about 2.4 standard deviations of the Gaussian) and a total of 30-60 thousand counts per PSF, the expected resolution would be at best $\frac{700}{\sqrt{60000}} \approx 3\ nm$, which is overall the error obtained from the fit. Indeed, the difference between each measurement is much larger than 3 nm, but no obvious trend or drift in the position appears. The recorded distance between the molecules reverts around the mean, whereas a random walk would lead to an increase of the distance. Hence, if there is any spatial diffusion, it is likely very limited, despite the many spectral jumps that occurred over the long measurement time.

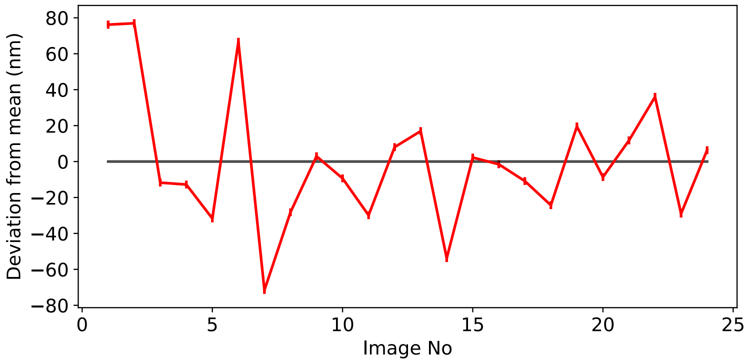

Supplementary Figure 15. Variations of the distance between the two molecules of Figure 14 extracted from 24 consecutive confocal images. The average error of the fit is just a few nm, but the measurements likely have a larger error due to spectral jumps occurring while recording the PSF. For all fitted data, the distance appears to revert around a mean value of 2.18 µm, and shows no clear trend. Hence, if there is any spatial diffusion, its extent is very limited. T = 2 K.

The substrate

Although our hBN flakes are relatively thick, as determined by AFM in section 1, we considered that the substrate could play a role for the spectral jumps. For some defects inside hBN, albeit for relatively thin flakes of 13 nm, the spectral stability has been reported to improve by coating the substrate with an alumina layer, to passivate the silica[14]. On one of our samples we found a flake that did not completely attach to the substrate and a part of it was free-standing at a large angle (Figure 17). Terrylene molecules had still condensed on the surface by vacuum sublimation, as can be observed on the part that is in focus in Figure 16a. The spectral time traces we recorded of two bright spots show that there are still spectral jumps present.

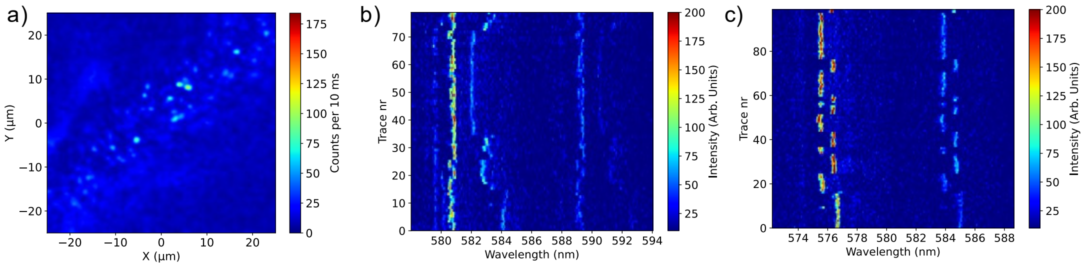

Supplementary Figure 16. a) Shows a fluorescence image from the part of an hBN flake that is not completely attached to the silicon/silica substrate, but was standing free at an angle with the substrate. We deposited terrylene molecules on this flake by vacuum sublimation. Only a part of the flake is in focus with the laser beam, along the diagonal of the figure. b) and c) show spectral traces of some terrylene molecules that are in focus, with their 0-0 ZPL and their strongest vibrational line around 248 cm^{-1}. Although there is no substrate in the vicinity of these molecules, large spectral jumps are still observed. The spectrum in b) is measured at the two dots in a) at coordinates (5.4, 8.0) and the spectrum in c) is measured at the single dot at coordinates (-6,-4) in a). T = 2 K.

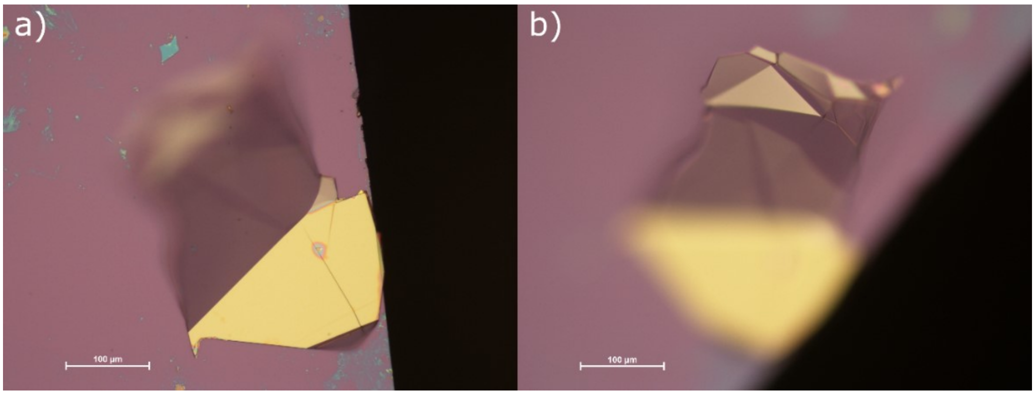

Supplementary Figure 17. a) Microscope image of the flake that is measured for Figure 16. The part in focus is in contact with the substrate, while in b) the non-attached part is in focus, above the surface of the substrate

Annealing

For single defects in CVD grown hBN it is reported that the width of the ZPL at room temperature was significantly improved by thermally annealing the hBN, with a clear improvement between an annealing temperature of 550 ˚C and 750 ˚C[15]. Similarly, we find that the spectral stability of the molecules improve significantly by annealing the flakes prior to deposition of the molecules. At 750 ˚C, maintained overnight for 12 hours, we obtain the best results. A subset of series of spectra are shown in Figure 18, which are complementary to the spectra shown in Figure 4 in the main text.

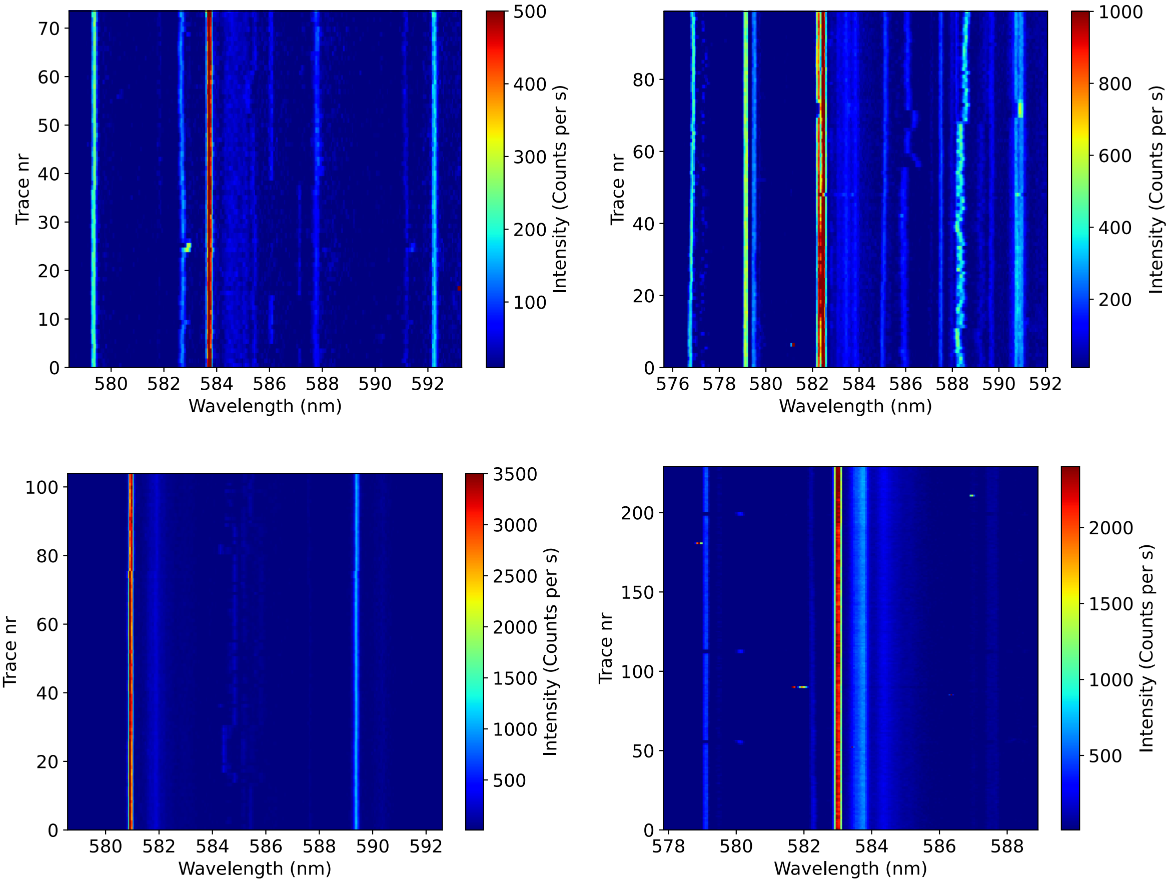

Supplementary Figure 18. Additional spectral series of annealed samples (750 ˚C for 12 hours). Some molecules are more isolated than others, but even in a small ensemble, as in the top right figure, most emitters do not move much. T = 2 K.

Supplementary Notes 5. Photon antibunching

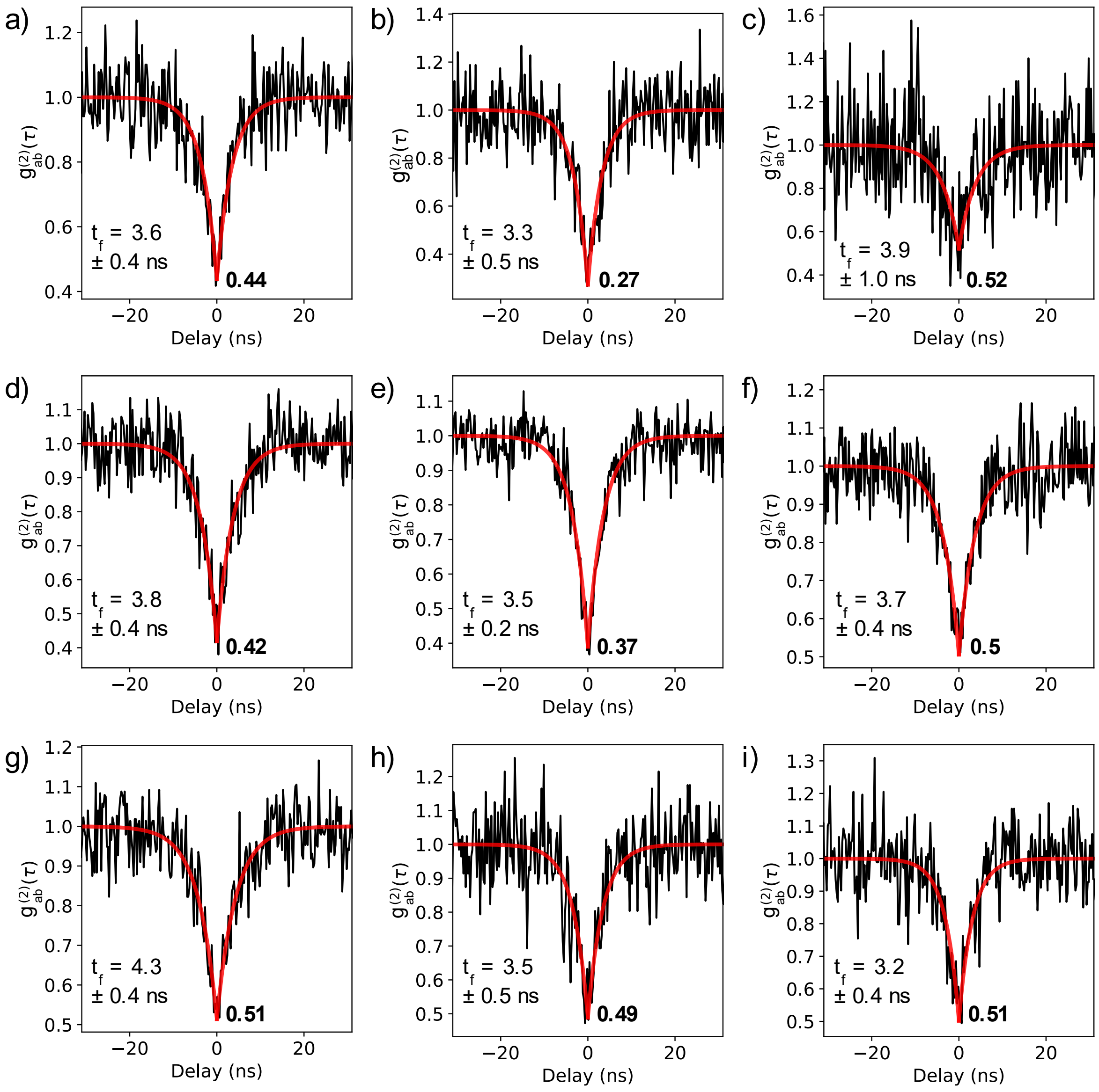

Supplementary Figure 19. Collection of antibunching measurements on the fluorescence signal of terrylene. Four out of nine antibunching curves display a contrast of less than 50 %, which in all cases is consistent with the measured background signal. The lifetimes obtained from the fit to $g_{ab}^{(2)}(\tau) = 1 - ce^{-|\tau|/\tau_f}$ are displayed on the bottom left and give an average of 3.6 ± 0.2 ns. The antibunching curve displayed in e) is also shown in the main text. The spectra of these nine molecules were measured as well, to confirm that the spectral signature corresponded to terrylene. T = 2 K.

Supplementary Notes 6. Low-temperature excitation spectra of the 0-0 ZPL

We use a tunable single-frequency laser, with a linewidth of about 1 MHz, to record excitation spectra of the 0-0 ZPL of single molecules. To find molecules, we position our laser's frequency around the peak of the inhomogeneous broadening, somewhere in between 581 and 583 nm. Furthermore, we set the laser intensity to be high enough to power-broaden 0-0 ZPLs in order to increase the number of molecules that emit fluorescence in a spectral range around the laser's frequency. Otherwise, the number of responsive molecules would be very low, due to the low concentration of molecules and the relatively large inhomogeneous broadening. To observe the responsive molecules, we record a fluorescence map of the flakes. An example is shown in Figure 20a. The bright pixels around (20, -25) belong to the molecule in Figure 5 in the main text.

Without positioning our laser on the molecule directly, we reduce the laser intensity to levels of a few W/cm^2 and start a scan of the laser. We do this to prevent the molecule from jumping away before we even start scanning the laser. After the first scan is completed, we move the laser spot to the position of the molecule. About half the time, we observe molecules with similar behaviour as shown in Figure 20b. A strong spectral diffusion, possibly in combination with spectral jumps, makes the molecule appear intermittently over the full scan range. From these type of molecules it is impossible to deduce a linewidth.

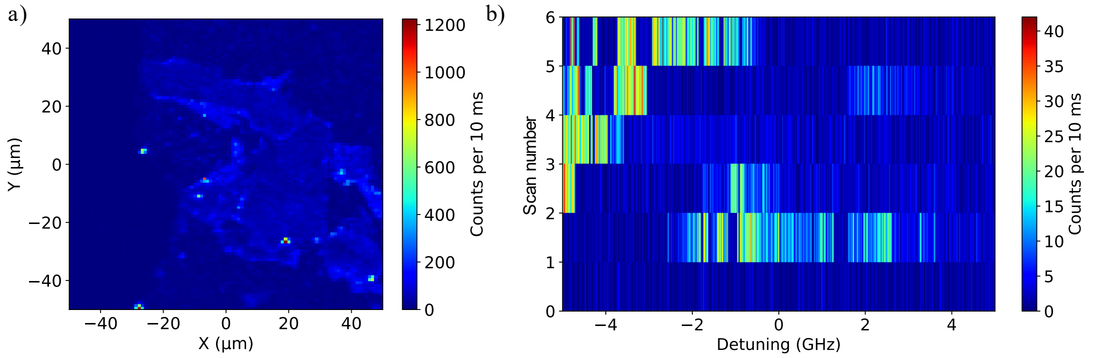

Supplementary Figure 20. a) Fluorescence map of hBN crystals at an excitation wavelength of 582.38 nm and a laser intensity of 350 W/cm^2. The bright spot around (20, -25) belongs to the molecule of Figure 5 in the main text. b) shows excitation spectra of a single molecule with very common behaviour for the 0-0 ZPL. The strong spectral diffusion and blinking makes it difficult to extract a linewidth. The sample was annealed at 750 ˚C for 12 hours and measurement temperature was 2 K.

For the remainder of the molecules it was possible to observe the 0-0 ZPL and fit a Lorentzian distribution to the fluorescence profile, as was the case for Figure 21d and Figure 22b. In some cases, a Gaussian distribution fits better, such as Figure 23b. In general, molecules can be followed for a limited time period, after which they jump out of the scan range. This occurs for the molecules in Figure 22a and 23a. Discrete jumps can also be observed within the scan range, such as in Figure 22a. This molecule has two Lorentzian distributions, spaced by a gap of 1.1 GHz. The two distributions have the same linewidth and the spacing remains intact after a spectral jump. Hence, the two lines are likely from the same molecule, which may be coupling to a fast-switching two-level system responsible for the line splitting.

In addition to discrete spectral jumps, molecules also show jittering spectral diffusion, which becomes stronger as the laser intensity is increased. Figure 21a,b and c show examples of such photo-induced spectral diffusion. Apart from expected power broadening of the linewidth, the amplitude of the spectral diffusion clearly increases as the laser intensity is raised by a factor 10 between the time series from a to c. Despite the spectral diffusion, the molecule was never lost in our limited scanning range of 10-20 GHz. Therefore we can do time-correlated single-photon counting and observe antibunching in the fluorescence emission (Figure 21e). As the molecule is driven resonantly by the laser, Rabi oscillations are expected to show up at these high excitation intensities. However, they are not clearly present, which means there is probably significant dephasing present or they are blurred by spectral diffusion. Despite absence of spectral diffusion for the molecule in Figure 5 in the main text, no Rabi oscillations appear in the antibunching histogram as well (Figure 24).

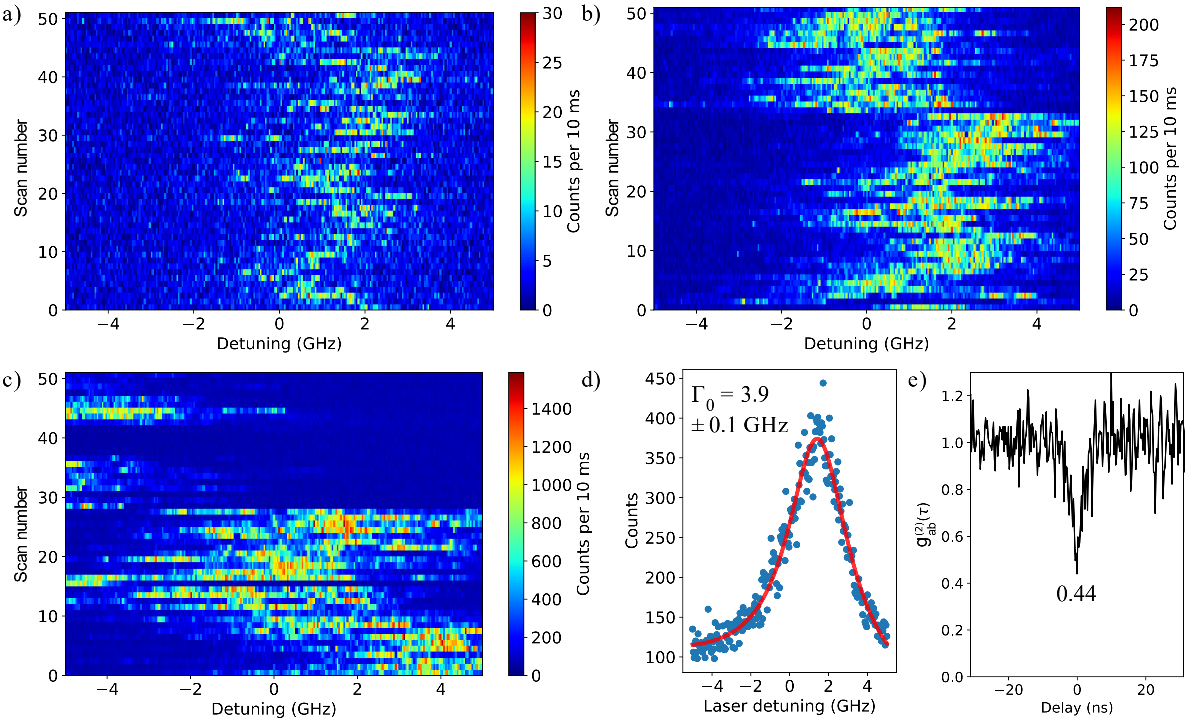

Supplementary Figure 21. a) Excitation spectra of the 0-0 ZPL of a single terrylene molecule measured at three different excitation intensities. The excitation intensity was 1 W/cm^2 in a), 14 W/cm^2 in b) and 250 W/cm^2 in c). d) We extracted the linewidth of the emitter by summing all excitation spectra in a), fitted to a Lorentzian distribution. In e) we recorded the correlation function of the resonance fluorescence, showing a dip at zero delay. The antibunching histogram was recorded with the high excitation power in c), which led to long blinking of the fluorescence signal due to spectral diffusion. The blinking, residual background and possibly limits of the instrument as a result of the short decay time, attributed to the relatively high dip at 0.44. We observe no Rabi oscillations, which is likely due to significant dephasing or spectral diffusion. The shorter decay time, compared to Figure 1b in the main text, is likely caused by stimulated emission. The sample was annealed at 750 ˚C for 12 hours and measurement temperature was 2 K.

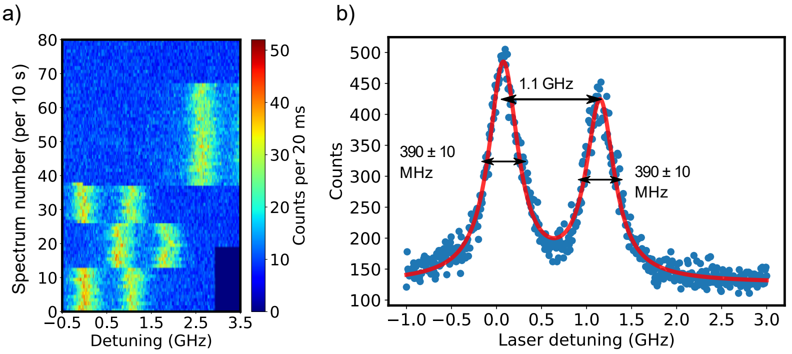

Supplementary Figure 22. a) Series of emission spectra of a molecule with a relatively narrow linewidth, found on a non-annealed flake. The two lines are likely related to coupling to a fast-switching two-level system, while slower jumps are related to slow-switching two-level systems. After about 10 minutes, the molecule jumped out of the scanned range. The complete series is composed of two individual scans that were shifted with respect to each other. The gap in the scan on the bottom right is the result of the shifted position of the two individual scans. b) shows the sum of the first ten excitation spectra, which is fit to a sum of two Lorentzian distributions. The linewidths of the two fits give the same number of 390 ± 10 MHz, about 8-9 times larger than the lifetime-limited linewidth.

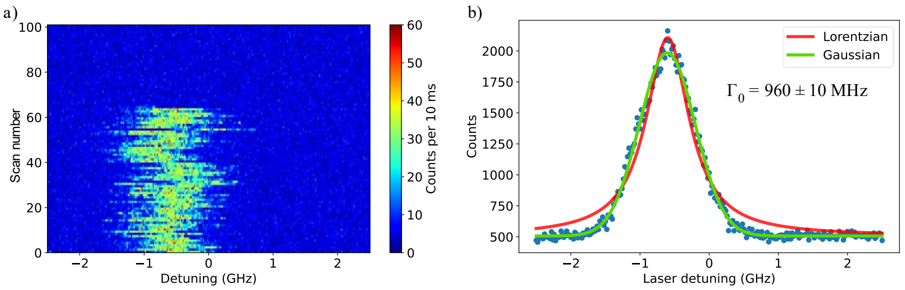

Supplementary Figure 23. a) Series of excitation spectra of a molecule at 581.96 nm, excited with a laser intensity of 11 W/cm^2. The molecule shows weak spectral diffusion, but jumps out of the scanned range at scan 65. b) Excitation spectrum of the 0-0 ZPL, which is derived from the sum of all excitation spectra in a). Due to the short tails, the spectral profile fits best to a Gaussian distribution (green line) with a linewidth of 960 ± 10 MHz. The red line shows the corresponding fit of a Lorentzian distribution. The sample was annealed at 750 ˚C for 12 hours and measurement temperature was 2 K.

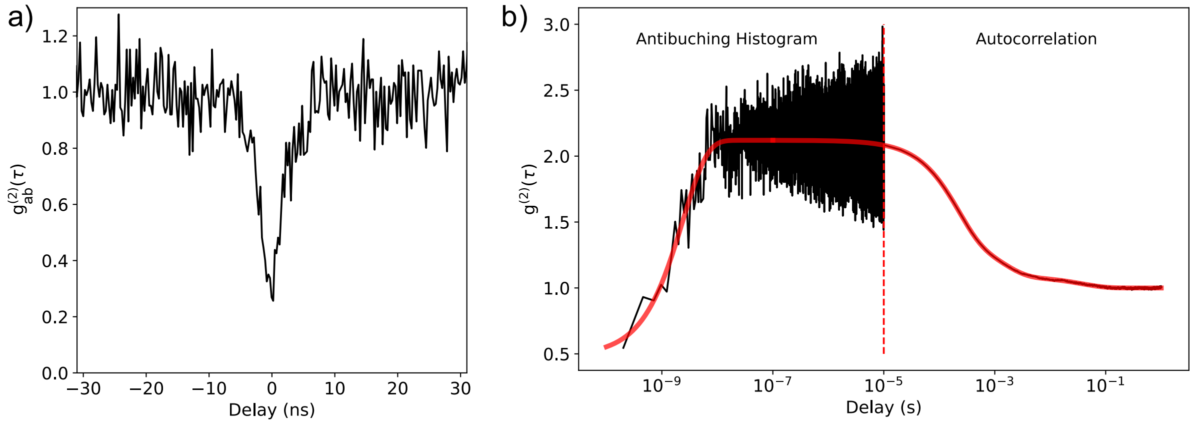

Supplementary Figure 24. a) Antibunching histogram for the resonantly excited molecule of Figure 5 in the main text, measured at 2 K. There is no clear presence of Rabi oscillations, while the laser intensity of 285 W/cm^2 was considerably higher than the saturation intensity of 105 ± 22 W/cm^2. As the linewidth is about 4.7 ± 0.3 GHz, the coherence time would be a minimum of 68 ± 4 ps, which is too short to be detected with our setup. The characteristic time of the exponential increase, starting from zero delay, of about 2.5 ns is significantly shorter than found for non-resonant excitation. This is likely the result of stimulated emission due to the relatively high intensity of the laser. b) Extended correlation function, constructed by a combination of the antibunching histogram in (a) (recorded up to 10 µs) and the autocorrelation function of the resonance fluorescence from the molecule, recorded with an integration time of 10 µs for a period of 60 seconds and calculated up to a time delay of 1 second using the function: $g^{(2)}(\tau) = \langle I(t)I(t+\tau)\rangle / \langle I(t)\rangle^2$. The addition of bunching due to triplet blinking leads to a rescaled normalization constant, reached at about 10-20 ns. The bunching of photons starts to decay significantly in the millisecond region. The left part of the figure was fitted to an exponential increase, while the right part of the figure is fitted to a three-exponential decay, together yielding the correlation function: $g^{(2)}(\tau) = 1 + C_1 e^{-\frac{t}{\tau_1}} + C_2 e^{-\frac{t}{\tau_2}} + C_3 e^{-\frac{t}{\tau_3}} - C_{ab} e^{-t/\tau_{ab}}$, with C_{ab} the contrast of the (rescaled) antibunching dip with decay rate τ_{ab} and C_1, C_2 and C_3 the contrasts of the exponentials with respective decay rates τ_1, τ_2 and τ_3. The time constants for the three exponents are 0.204 ms, 1.344 ms and 37.23 ms. The longest time constant is significantly longer than typical triplet lifetimes for terrylene and thus could be the result of a two-level system(s). These long blinking times were also observed in the quantum jumps of Figure 5b in the main text and the tail of the dark time histogram in Figure 5e of the main text.

Supplementary Notes 7. Impurity emitters on hBN

We set up a control experiment with hBN flakes that were spin-coated with toluene with no dye dissolved or flakes which have undergone no treatment at all. On the untreated hBN flakes no narrow emitters were found. However, on the flakes that were spin-coated with toluene several narrow emitters could be found. Frequently occurring emitters clearly have the vibrational fingerprint (Figure 25) of a well-known impurity that is typically found in organic and polymer layers[16,17], sometimes denoted as molecule X. In PMMA matrices these emitters were found over a broad range of 1.9 eV (650 nm) up to 2.2 eV (570 nm). On hBN we find spectra characteristic of molecule X or compounds of its family over a wavelength range from 618 nm up to 640 nm (Figure 26), which is similar as the distribution found in polymers[17].

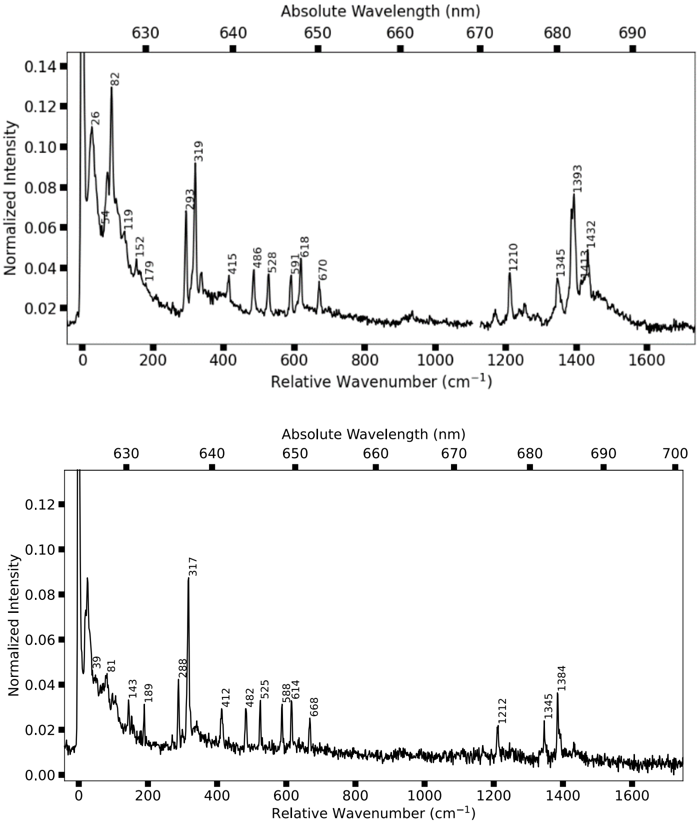

Supplementary Figure 25. Two spectra of the impurity molecules that we find on samples prepared by spin-coating with a toluene solution. Some of the peak's intensities vary significantly from emitter to emitter. The vertical axis has been cut for clarity.

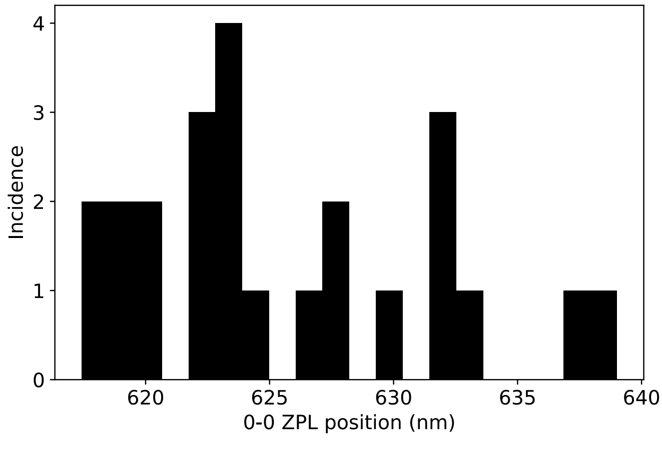

Supplementary Figure 26. Histogram of the 0-0 ZPL positions of the same impurity emitters as in Figure 25. A total of 24 of these emitters were found in our experiments.

Supplementary References

1. Zhang, Y. *et al.* Probing Carrier Transport and Structure-Property Relationship of Highly Ordered Organic Semiconductors at the Two-Dimensional Limit. *Phys. Rev. Lett.* **116**, 016602 (2016).
2. Park, B. *et al.* Anomalous Ambipolar Transport of Organic Semiconducting Crystals via Control of Molecular Packing Structures. *ACS Appl. Mater. Interfaces* **9**, 27839–27846 (2017).
3. Palewska, K. *et al.* Total Luminescence Spectroscopy of Terrylene in Low-Temperature Shpol'skii Matrixes. *J. Phys. Chem.* **99**, 16835–16841 (1995).
4. Deperasińska, I. & Kozankiewicz, B. Non-planar distortion of terrylene molecules in a naphthalene crystal. *Chem. Phys. Lett.* **684**, 208–211 (2017).
5. Kurdyumov, A. V., Solozhenko, V. L. & Zelyavski, W. B. Lattice Parameters of Boron Nitride Polymorphous Modifications as a Function of Their Crystal-Structure Perfection. *J. Appl. Crystallogr.* **28**, 540–545 (1995).
6. Deperasińska, I., Kozankiewicz, B., Biktchantaev, I. & Sepioł, J. Anomalous Fluorescence of Terrylene in Neon Matrix. *J. Phys. Chem. A* **105**, 810–814 (2001).
7. Hod, O. Graphite and Hexagonal Boron-Nitride have the Same Interlayer Distance. Why? *J. Chem. Theory Comput.* **8**, 1360–1369 (2012).
8. Kim, S. M. *et al.* Synthesis of Patched or Stacked Graphene and hBN Flakes: A Route to Hybrid Structure Discovery. *Nano Lett.* **13**, 933–941 (2013).
9. Lee, W. H. *et al.* Surface-Directed Molecular Assembly of Pentacene on Monolayer Graphene for High-Performance Organic Transistors. *J. Am. Chem. Soc.* **133**, 4447–4454 (2011).
10. Cassabois, G., Valvin, P. & Gil, B. Hexagonal boron nitride is an indirect bandgap semiconductor. *Nat. Photonics* **10**, 262–266 (2016).
11. Navarro, P. *et al.* Electron Energy Loss of Terrylene Deposited on Au(111): Vibrational and Electronic Spectroscopy. *J. Phys. Chem. C* **119**, 277–283 (2015).
12. Tchénio, P., Myers, A. B. & Moerner, W. E. Optical studies of single terrylene molecules in polyethylene. *J. Lumin.* **56**, 1–14 (1993).
13. Nicolet, A., Kol'chenko, M. A., Kozankiewicz, B. & Orrit, M. Intermolecular intersystem crossing in single-molecule spectroscopy: Terrylene in anthracene crystal. *J. Chem. Phys.* **124**, 164711 (2006).

14. Li, X. *et al.* Nonmagnetic Quantum Emitters in Boron Nitride with Ultranarrow and Sideband-Free Emission Spectra. *ACS Nano* **11**, 6652–6660 (2017).

15. Li, C. *et al.* Purification of single-photon emission from hBN using post-processing treatments. *Nanophotonics* **8**, 2049–2055 (2019).

16. Fleury, L. *et al.* Single Molecule Spectra of an Impurity Found in N-Hexadecane and Polyethylene. *Mol. Cryst. Liq. Cryst. Sci. Technol. Sect. Mol. Cryst. Liq. Cryst.* **283**, 81–87 (1996).

17. Neumann, A., Lindlau, J., Thoms, S., Basché, T. & Högele, A. Accidental Contamination of Substrates and Polymer Films by Organic Quantum Emitters. *Nano Lett.* **19**, 3207–3213 (2019).
